# Effects of Dispositional Affect on the N400: Language Processing and Socially Situated Context

**DOI:** 10.3389/fpsyg.2021.566894

**Published:** 2021-03-31

**Authors:** Veena D. Dwivedi, Janahan Selvanayagam

**Affiliations:** ^1^Department of Psychology and Centre for Neuroscience, Brock University, St. Catharines, ON, Canada; ^2^Department of Physiology and Pharmacology, University of Western Ontario, London, ON, Canada

**Keywords:** N400, dispositional affect, conceptual semantics, sentence comprehension, mood/emotion, global-local

## Abstract

We examined whether the N400 Event-Related Potential (ERP) component would be modulated by dispositional affect during sentence processing. In this study, 33 participants read sentences manipulated by direct object type (congruent vs. incongruent) and object determiner type (definite vs. demonstrative). We were particularly interested in sentences of the form: (i) *The connoisseur tasted the* ***wine*** *on the tour* vs. (ii) *The connoisseur tasted the #* ***roof*** *…* We expected that processing incongruent direct objects (#*roof*) vs. congruent objects (*wine*) would elicit N400 effects. Previous ERP language experiments have shown that participants in (induced) positive and negative moods were differentially sensitive to semantic anomaly, resulting in different N400 effects. Presently, we ask whether individual dispositional affect scores (as measured by the Positive and Negative Affect Schedule; PANAS) would modulate N400 effects as shown previously. Namely, previous results showed larger N400 effects associated with happy moods and attenuated amplitudes associated with sad moods. Results revealed significant N400 effects, driven by *the #roof* vs. *the wine*, where larger amplitude differences were found for individuals showing smaller negative affect (NA) scores, thus partially replicating previous findings. We discuss our results in terms of theories of local (lexical) inhibition, such that low NA promotes stronger lexico-semantic links in sentences. Finally, our results support accounts of language processing that include social and biological characteristics of individuals during real-time sentence comprehension.

## Introduction

It is well established that individuals differ in their responses to identical stimuli. Whereas one individual perceives, and interprets a glass filled at the 50% mark as half-full, another perceives, and interprets it as half-empty. A common-sense explanation for these differences is that the personality and/or mood of the perceiver plays a role in the above-mentioned interpretive possibilities.

The idea that the emotional mood of the perceiver may influence interpretation is supported by research in cognitive psychology (see, inter alia, Ashby et al., 1999; Gasper and Clore, 2002; Fredrickson and Branigan, 2005; Dale and Arnell, 2010). For example, it has been claimed that positive moods are associated with greater global processing, such that individuals are more sensitive to top-down contextual knowledge. In contrast, negative moods tend to be associated with cognitive styles characterized by item specific processing, where attention is more focused on details (Kimchi and Palmer, 1982; Schwarz, 2002; Rowe et al., 2007). With respect to mood effects on language processing, Chwilla et al. (2011) conducted a series of studies using Event-Related Potential (ERP) methods (Vissers et al., 2010, 2013; see also Federmeier et al., 2001; van Berkum et al., 2013). They used a paradigm where participants were induced into happy or sad emotional moods by watching either happy or sad movies (e.g., short clips of either *Happy Feet* or *Sophie’s Choice*). Their studies showed that participants in happy moods exhibited modulation of the language-related ERP effects of interest, whereas sad moods attenuated ERP responses. Relevant to our current study, Chwilla et al. (2011) focused on the N400 component, a negative-going waveform that peaks approximately 400 ms after a word, which does not match previous sentential context in terms of lexico-semantic fit (Kutas and Hillyard, 1980, 1984). In that work, they examined sentences of the form “*The pillows are stuffed with* ***feathers*** *….* vs. “*The pillows are stuffed with* ***books***…” and showed that the latter sentence with the low-cloze fragment showed larger N400 effects for happy vs. sad participants. They interpreted their findings as consistent with literature on mood and cognition (see above, as well as Schwarz and Clore, 2007), where happy individuals were more sensitive to global features of stimuli. That is, global features of sentence interpretation are associated with stereotypical event knowledge, also called script or schemata (cf. Schank and Abelson, 1977; St. George et al., 1994; Chwilla and Kolk, 2005). Finally, they concluded that their ERP results supported the recent shift in cognitive neuroscience that views cognition as “hot” (i.e., not independent of mood) vs. “cold.”

In the present work, we build on the findings above, as well as recent work in our own lab (Selvanayagam et al., 2019) and ask whether the related construct of dispositional affect would modulate sentence interpretation as measured via the N400 component.

In contrast to mood, which can fluctuate according to situation, dispositional affect reflects the stability across time and situation of individuals to view their world with approach-oriented positive affect (PA), or avoidance-oriented negative affect (NA). That is, it is a personality trait, reflective of individual differences (Watson and Clark, 1984; Staw and Ross, 1985; Levin and Stokes, 1989).

As such, we can consider dispositional affect of an individual as a socially relevant and arguably biological characteristic of an individual during language comprehension. The Positive and Negative Affect Schedule (PANAS; Watson et al., 1988) indexes trait and state features of affect and mood. Given that it has been shown that there are individual differences in temperaments that can be more or less susceptible to mood induction (Larsen and Ketelaar, 1989, 1991; Brief et al., 1995), we ask whether dispositional affect, as measured by the PANAS, modulated N400 responses to sentences. In a preliminary investigation in our lab (Selvanayagam et al., 2019), we showed that the P300 effect was indeed influenced by affect. Results were consistent with the view that less positive individuals, measured via the PANAS, were less sensitive to global heuristic cues of meaning.

In the current experiment, we focus on the N400 ERP component. We examined neural responses to words that were either congruent or incongruent with sentential context. That is, we contrasted sentences of the form *The connoisseur tasted the* ***wine*** *during the tour* vs. *The connoisseur tasted the #* ***roof*** ^1^ *during the tour*. The direct object in the latter sentence clearly violates our real-world expectations of what possible objects of tasting might be. As such, the critical word ***roof*** should elicit an N400 effect vs. its control ***wine***. Our predictions regarding dispositional affect, prima facie, would be to replicate the findings of the induced mood participants in Chwilla et al. (2011). That is, N400 effects would be larger for individuals exhibiting higher PA scores, given findings of larger N400 effects for happy participants. Furthermore, N400 effects would be smaller for individuals with higher NA scores, mirroring previous findings of attenuated N400 effects for sad participants. These findings are expected on the cognitive view that positive participants should be more sensitive to lexico-semantic cues regarding global sentence meaning (also called heuristics, Townsend and Bever, 2001; Ferreira, 2003; Dwivedi, 2013) vs. negative participants, who are not. When a word does not match its lexico-semantic context, the greater sensitivity of positive (vs. negative) participants to this mismatch should therefore elicit larger N400 effects.

As such, correlational analyses will be conducted between N400 amplitudes and positive and negative affect scores. We predict (i) a significant positive correlation between N400 amplitude and positive affect scores as well as (ii) a significant negative correlation between the size of the N400 effect and negative affect scores of our participants.

Next, we note here that a separate condition was included in this experiment to ask a question independent of dispositional affect and is discussed in detail elsewhere (Dwivedi and Selvanayagam, 2020a,b). Briefly, we wanted to know whether neural responses to lexico-semantic incongruency differed from those elicited via discourse semantic violations. We did so by examining “double violations” (Hagoort, 2003). As such, the other independent variable we examined was determiner type at object position, such as The connoisseur tasted *that ^2^ *#* ***roof*** *…* (without previous context, use of *that* results in presuppositional violation). In terms of our present study, the double violation condition might show the largest N400 effect, and if so, it too would correlate with positive and negative affect scores as above. See Table 1 for list of conditions.

**Table 1:** Critical conditions with example stimuli.

		Object type	
		Congruent	Incongruent
Object Determiner	Definite	𝑇ℎ𝑒 𝑐𝑜𝑛𝑛𝑜𝑖𝑠𝑠𝑒𝑢𝑟 𝑡𝑎𝑠𝑡𝑒𝑑 **the 𝒘𝒊𝒏𝒆** 𝑑𝑢𝑟𝑖𝑛𝑔 𝑡ℎ𝑒 𝑡𝑜𝑢𝑟.	𝑇ℎ𝑒 𝑐𝑜𝑛𝑛𝑜𝑖𝑠𝑠𝑒𝑢𝑟 𝑡𝑎𝑠𝑡𝑒𝑑 **the 𝒓𝒐𝒐𝒇** 𝑑𝑢𝑟𝑖𝑛𝑔 𝑡ℎ𝑒 𝑡𝑜𝑢𝑟.
	Demonstrative	𝑇ℎ𝑒 𝑐𝑜𝑛𝑛𝑜𝑖𝑠𝑠𝑒𝑢𝑟 𝑡𝑎𝑠𝑡𝑒𝑑 **that 𝒘𝒊𝒏𝒆** 𝑑𝑢𝑟𝑖𝑛𝑔 𝑡ℎ𝑒 𝑡𝑜𝑢𝑟.	𝑇ℎ𝑒 𝑐𝑜𝑛𝑛𝑜𝑖𝑠𝑠𝑒𝑢𝑟 𝑡𝑎𝑠𝑡𝑒𝑑 **that 𝒓𝒐𝒐𝒇** 𝑑𝑢𝑟𝑖𝑛𝑔 𝑡ℎ𝑒 𝑡𝑜𝑢𝑟.

## Methods

### Participants

Thirty seven Brock University undergraduates were recruited and either paid for their participation or received partial course credit. All participants were native, monolingual speakers of English, had normal or corrected-to-normal vision and were right-handed, as assessed by the Handedness Inventory. No participants reported any neurological impairment, history of neurological trauma, or use of neuroleptics.

Four participants with comprehension question accuracy for filler items (discussed below) at less than 85% were excluded from analysis leaving 33 eligible participants (25 females; mean age = 19.6; ranging from 18 to 25).

This study received ethics approval from the Brock University Bioscience Research Ethics Board (BREB) prior to the commencement of the experiment (REB 13-282). Written, informed consent was received from all participants prior to their participation in the experiment.

### Materials

160 critical items (adapted from Dwivedi and Gibson, 2017) were presented in four conditions (see Table 1) counterbalanced across four lists. All sentences in this experiment were simple active sentences, using SUBJECT VERB OBJECT word order, followed by a prepositional phrase. Sentences varied according to two factors: object type (congruent vs. incongruent object) and determiner type (definite vs. demonstrative). All subjects were animate (e.g., *connoisseur, kid*) and preceded by the definite determiner *the*. An active, past-tense verb followed the subject (e.g., *tasted, climbed*). All direct objects were inanimate (e.g., *wine, roof*) and were either congruent with sentence context in the control conditions (e.g., *connoisseur – tasted – **wine**, kid – climbed – **tree***) or incongruent with sentential context (e.g., *connoisseur – tasted – **roof**, kid – climbed – **jade***). Direct objects were not repeated and were matched for word length item by item (e.g., *wine* vs. #*roof*). Next, word frequency for direct objects in congruent vs. incongruent conditions was controlled for, where log word frequencies (SUBTLEX-US database; see Brysbaert and New, 2009) indicated no significant difference, *t*(159) = 0.63, *p* = 0.533. Also, the direct object was never the final word in the sentence. Sentences ended with prepositional phrases that (crucially) did not alter the interpretation of the direct object. Instead, these phrases served to modify the event by referring to time (e.g., *in the morning*), manner (e.g., *with difficulty*), and instrument (e.g., *with a pen*). Comprehension questions did not follow presentation of critical trials (stimuli available upon request).

To reduce predictability, 170 filler sentences were included, of varying syntactic and semantic structure.^3^ These sentences were 6–10 words in length and a subset of these (125 items, 38% of all trials) were followed by superficial Yes/No or True/False comprehension questions.

### Offline Plausibility Ratings

We evaluated the plausibility of our critical materials by conducting a norming study using Qualtrics software, Version (March 2020) of the Qualtrics Research Suite (Qualtrics, 2020). Critical and filler sentences were rated in this web-based study according to plausibility on a scale from 0 (very implausible) to 5 (neutral) to 10 (very plausible), in steps of 0.1. The 160 critical items were presented in eight pseudorandomized, counterbalanced lists such that half of the critical items were presented in each list and each participant only saw each item once. 80 filler items were presented in all lists, for a total 160 items in each list. 34 participants completed the study, of which 30 met the eligibility criteria described above (as outlined in Section “Participants”). 10 participants were excluded for having a mean plausibility rating lower than seven on filler items (all of which were perfectly plausible). Data from the remaining 20 participants (20 females; mean age = 18.65; ranging from 18 to 25) were used to calculate plausibility ratings. A few trials had to be excluded due to software error (<0.3% of trials). Mean plausibility ratings for the critical conditions were: congruent definite (*the wine*; *M* = 8.43, *SD* = 0.85), congruent demonstrative (*that* wine; *M* = 7.99, *SD* = 0.99), incongruent definite (*the roof*; *M* = 2.25, *SD* = 2.10); incongruent demonstrative (*that roof*; *M* = 2.05, *SD* = 2.07). An ANOVA was conducted on the mean plausibility ratings with the independent variables of object type (congruent vs. incongruent), determiner type (definite vs. demonstrative). Significant main effects of object type, *F*(1, 19) = 220.7, *MSE* = 3.33, *p* < 0.001, *ƞ_p_*^2^ = 0.920, and determiner type, *F*(1, 19) = 6.00, *MSE* = 0.34, *p* = 0.024, *ƞ_p_*^2^ = 0.240 were observed. However, no significant interaction of object and determiner type was observed, *F*(1, 19) = 1.60, *MSE* = 0.17, *p* = 0.221, *ƞ_p_*^2^ = 0.078. Overall, these results confirm the intended readings regarding congruent vs. incongruent sentences.

### Electrophysiological Measures

Electroencephalographic (EEG) recordings were made using a 64-channel Active Two BioSemi system (BioSemi, Amsterdam). Data were sampled at a rate of 512 Hz and digitized with a 24-bit analog-to-digital converter. EEG data were preprocessed offline using EMSE v5.5.1 software (Cortech Solutions, 2013). Two infinite impulse response filters were applied at 12 db/octave: a bandpass filter from 0.1 to 100 Hz used to remove high and low frequency noise and a bandstop filter from 59 to 61 Hz used to remove 60 Hz noise. All electrodes were re-referenced to the averaged mastoids for analysis. Prior to segmentation, eye movements artifacts and blinks were filtered from the data using a spatial ocular artifact correction algorithm (Pflieger, 2001). Due to equipment malfunction, data from electrode Fp1 was lost in some participants. A spatial interpolation filter (Cortech Solutions, 2013) was applied for this electrode, for all participants. Manual artifact rejection was applied. Epochs were created from an interval 200 ms prior to stimulus onset to 1,200 ms after stimulus onset.

### Procedure

**Figure 1:**
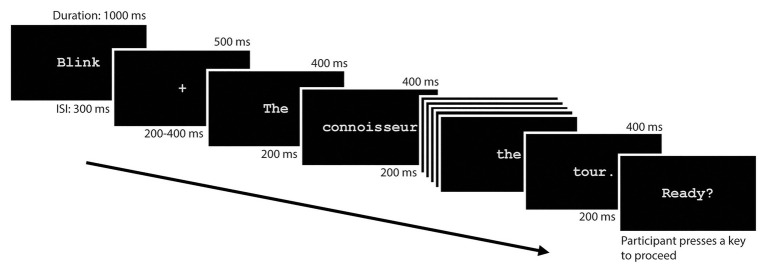
Condensed sample trial for the current paradigm. Time values above the screen represent the duration of stimulus presentation on screen whereas time values below the screen represent the inter-stimulus interval (ISI). The “Ready?” slide requires participant input to proceed and is sometimes preceded by a comprehension question.

Participants were tested individually in one session of approximately 3 h. In each session, participants completed a short questionnaire regarding reading habits, a handedness inventory (Briggs and Nebes, 1975), and the PANAS (Watson et al., 1988) before the application of the electrodes. PANAS consists of 20 items (10 positive items, e.g., interested, excited, and 10 negative items, e.g., distressed, upset) for which the participants provided a response on a five-point Likert scale indicating the extent to which they felt this way (1 = “Very slightly or not at all,” 5 = “Extremely”; see Watson et al., 1988 for further details). Following a practice session of eight trials, each participant completed the experimental trials in six blocks of 55 trials, with rest periods between each block. Each participant saw one of four pseudorandomized, counterbalanced lists consisting of 330 items. The pseudorandomized lists were created using the Mix utility (van Casteren and Davis, 2006) such that the first three items and last two items of each block were always filler sentences; no more than two critical items were presented sequentially and items from the same condition were never presented sequentially. Using E-Prime 2.0 software (Psychology Software Tools, Pittsburgh, PA), sentences were presented in the center of the computer monitor (screen size 50.8 cm) in light gray, 18-point Courier New font on a black background at a viewing distance of approximately 70 cm. See Figure 1 for a sample trial procedure (Psychology Software Tools, Inc. [E-Prime 2.0], 2012).

Each trial sentence began with the participant being prompted to press a button on the response pad, then the word “Blink” was presented for 1,000 ms, followed by a fixation cross (+) for 500 ms. After a variable inter-trial interval lasting between 200 and 400 ms, sentences were presented word-by-word in serial visual presentation mode with a stimulus onset asynchrony (SOA) of 600 ms and an inter-stimulus interval (ISI) of 200 ms. 125 filler items were followed by comprehension questions after the last word of the sentence, to which participants were asked to press a “1” or “2” key corresponding to answers on the screen using the response pad. Response time and accuracy was recorded for each response. The next trial began following another inter-trial interval lasting between 500 and 1,000 ms.

## Results

### Behavioral Analyses

#### Filler Comprehension Questions

Comprehension rates for questions at filler conditions were at 95.20% (*SD* = 2.75%), indicating that participants were indeed paying attention to sentence materials.

### Electrophysiological Analyses

**Figure 2:**
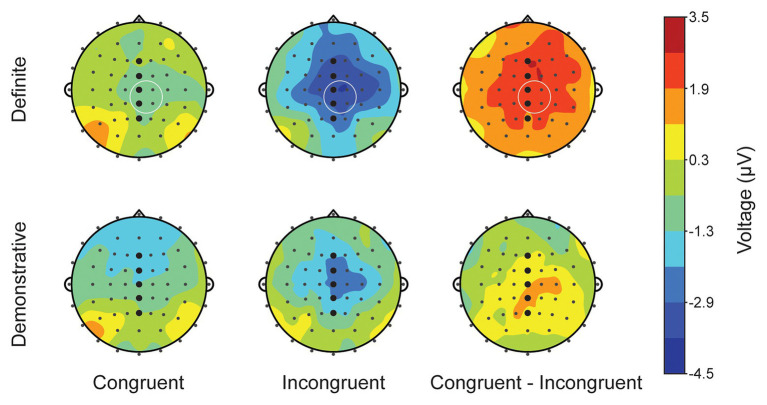
Topographic plots of mean amplitude (μV) 300–500 ms after stimulus onset at CW for definite (top) and demonstrative (bottom) conditions. Black dots indicate scalp position of electrodes. Bolded black dots indicate midline electrode positions, used for ANOVA (see Section “N400 at the critical word”); white circles indicate the region of interest (ROI) used in correlational analyses (see Section “Correlational analyses”).

**Figure 3:**
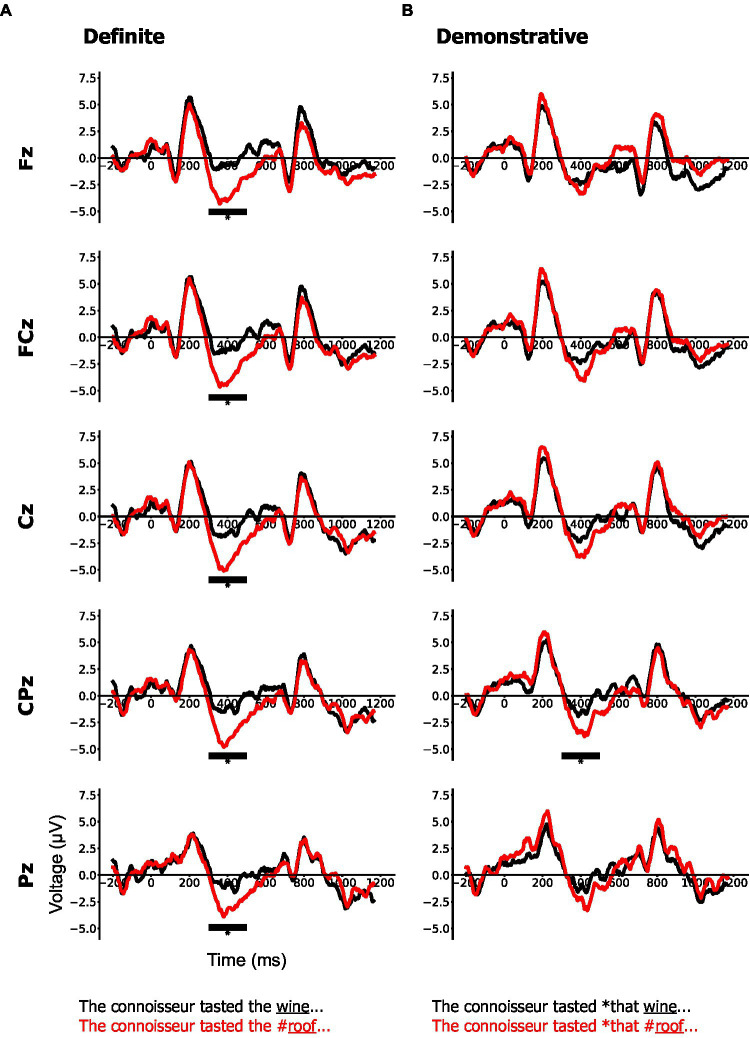
Grand average event-related potential (ERP) waveforms for object type condition: congruent (black) and incongruent (red) separately for definite **(A)** and demonstrative **(B)** conditions at five midline electrodes (Fz, FCz, Cz, CPz, and Pz) time-locked to the onset of the critical word (*wine/#roof*). Asterisks * indicate significant amplitude differences (see Section “N400 at the critical word”).

Figure 2 shows a topographic map for 33 participants, in the typical N400 time range (300–500 ms) after critical word (*wine/#roof*) onset. It shows a large, broadly distributed N400 effect with slight right lateralization in the definite condition, and a smaller N400 effect, constrained to centroparietal sites, also with slight right lateralization, in the demonstrative condition. Given this broad (and typical, see Federmeier and Kutas, 1999) distribution, we focus analyses at midline sites (see grand average ERPs, Figure 3).

#### N400 at the Critical Word

A repeated measures ANOVA was conducted for midline electrode sites (Fz, FCz, Cz, CPz, and Pz) at the critical word (*wine* vs. *#roof*) on mean voltage in the traditional N400 time window (300–500 ms) using SPSS (IBM Corp, 2018) statistical software (see Table 2). We employed the Greenhouse-Geisser (Greenhouse and Geisser, 1959) non-sphericity correction for effects with more than one degree of freedom in the numerator. Following convention, unadjusted degrees of freedom are reported, along with the Greenhouse-Geisser epsilon value (*ε*) and adjusted *p* value. Mean square error values reported are those corresponding to the Greenhouse-Geisser correction. Partial eta squared (*η_p_*^2^) is reported as a measure of effect size and *post hoc* comparisons are Bonferroni corrected.

**Table 2:** *F*-values for midline analyses of Object and Determiner type at critical word for time window 300–500 ms.

Effect (df)	*F*	*p*	*MSE*	*η_p_*^2^
O (1, 32)	30.982	<0.001^*^	13.727	0.492
D (1, 32)	1.362	0.252	18.283	0.041
E (4, 128)	5.580	0.004^*^	5.655	0.148
O * D (1, 32)	5.047	0.032^*^	15.920	0.136
O * E (4, 128)	1.270	0.290	2.038	0.038
D * E (4, 128)	3.346	0.026^*^	1.843	0.095
O * D * E (4, 128)	3.221	0.047^*^	1.764	0.091

O, object type; D, determiner type; and E, electrode site.

* < 0.05.

Here, we observed the incongruent condition was significantly more negative than its control in the definite condition at all midline electrode sites (*the wine/#roof*; Δ = 1.76–2.65 μV, all values of *p* < 0.001; see Figure 3A), whereas the incongruent condition was significantly more negative than its control in the demonstrative condition only at electrode site CPz (*that wine/#roof*; Δ = 1.42 μV, *p* = 0.006; all other values of *p* > 0.05; see Figure 3B). Thus, a large, broadly distributed N400 effect was observed for a semantic anomaly preceded by the definite determiner *the*, whereas a heavily attenuated N400 effect restricted to one site was observed for a semantic anomaly preceded by the demonstrative determiner *that*.

#### Correlational Analyses

To investigate the relationship between N400 amplitude and dispositional affect, we computed difference scores by subtracting the mean amplitude at incongruent conditions from congruent conditions in the definite condition, which had elicited a robust N400 effect (e.g., *the wine – the #roof*). Based on visual inspection of the N400 effect (see Figure 2), we chose a region of interest (ROI) consisting of right lateralized centroparietal electrode sites (Cz, C2, CPz, and CP2) where the effect was maximal, a region consistent with and typical of N400 scalp topography (Federmeier and Kutas, 1999; Kutas and Federmeier, 2011). We computed the difference in the mean amplitude at the average of these electrode sites at 300–500 ms following stimulus onset. These difference scores were correlated with PA (*M* = 27.61; *SD* = 6.89; ranging from 14 to 35) and Negative Affect (NA; *M* = 15.73; *SD* = 4.71; ranging from 10 to 28) scores.

**Figure 4:**
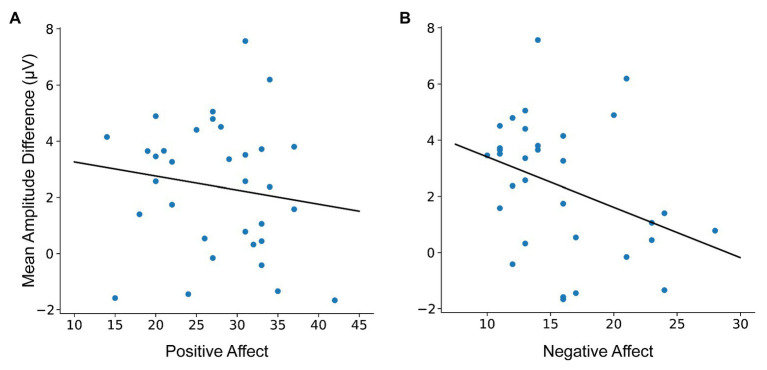
Scatterplot of mean N400 amplitude difference in the definite condition (*the wine* – *the #roof*) with **(A)** Positive Affect (PA) and **(B)** Negative Affect (NA) at the right lateralized centroparietal ROI (Cz, C2, CPz, and CP2).

We did not observe a significant correlation between PA scores and N400 amplitudes (see Figure 4A), *r*(31) = −0.15, *p* = 0.419.^4^ We observed a moderate negative correlation between NA scores and N400 amplitude (see Figure 4B), *r*(31) = −0.36, *p* = 0.041. That is, participants with larger NA scores had smaller N400 amplitudes (conversely, participants with smaller NA scores had larger N400 amplitudes).

## Discussion

In the present study, we examined whether N400 responses to incongruent objects in sentences would be modulated by dispositional affect scores. Based on previous work on mood and language processing, we predicted (i) a significant positive correlation between N400 amplitude and positive affect scores, in addition to (ii) a negative correlation for this ERP component and negative affect scores. Our second prediction was borne out in this study. That is, N400 effects at incongruent *#the roof* vs. *the wine*, were modulated, such that more negative participants had smaller N400 amplitudes, whereas no correlation was found with positive affect. In a separate question, we also examined how neural responses to sentences containing “double violations,” as in *that #roof might differ from those with single violations. Results revealed an attenuated N400 response. This question and results are addressed in separate work (Dwivedi and Selvanayagam, 2020a,b) and we do not further discuss that question, to not detract from the issue at hand.

Below, we address the cognitive significance of our findings regarding dispositional affect and sentence perception. Specifically, we do so relying on previous work in emotion literature (Schwarz and Clore, 2007), which indicated that positive affect promotes global processing (focusing on the forest vs. the trees) vs. negative affect, which promoted more local processing (focusing on the trees vs. the forest).

### Dispositional Affect and Conceptual Event Semantic Processing

To reiterate, we found no correlation with positive affect and N400 amplitude, in contrast to a significant negative correlation with negative affect. Previously, Chwilla et al. (2011) found that participants induced into happy moods produced large N400 effects, whereas those in sad moods produced attenuated effects. A corollary of the latter finding is that low negative affect individuals produce larger N400 effects – exactly what we found. We interpret the cognitive significance of our result to mean that low negative affect can facilitate contextual or heuristic processing, which we discuss in further detail below.

Real-time sentence comprehension necessarily involves global context/event knowledge for interpretation (Barton and Sanford, 1993; Zwaan and Radvansky, 1998; Ferretti et al., 2007; Dwivedi et al., 2018). We claim that low negative affect promotes local associations between words in sentences and in this way facilitates sensitivity to contextual knowledge (Chwilla et al., 1998). Here, we draw on work by Huntsinger et al. (2010, p. 725) who indicate that “a global focus should result either from having an accessible global orientation empowered by positive affect, or a local orientation inhibited by negative affect.” We draw on this logic and reason that if negative affect inhibits a local orientation, then lower negative affect would lessen that inhibition. Less inhibition between words would allow for more spreading activation and build stronger lexical expectations between words during sentence comprehension (Kamide et al., 2003; Madden and Zwaan, 2003; McRae et al., 2005). A natural outcome of this increased spreading activation between words would be stronger lexical expectations during sentence comprehension (Collins and Loftus, 1975; Metusalem et al., 2012). In this way, larger N400 amplitudes are elicited for low negative affect individuals when lexical expectations are violated. In contrast, a high negative affect individual would strongly inhibit local lexical meaning, yielding weaker links between words in a sentence and therefore exhibit less sensitivity to incongruent meaning – thereby producing attenuated N400 effects.

We speculate here that the account above could explain the lack of an effect found with positive affect in our experiment, vs. what was found previously in Chwilla et al. (2011). We claim that differences in experimental stimuli design between our work and Chwilla et al. (2011) resulted in different processing strategies, which would be differentially sensitive to affect. Recall that sentence stimuli of Chwilla et al. (2011) consisted of high-cloze vs. low-cloze word manipulations (e.g., *stuffing pillows with feathers* vs. *stuffing pillows with #books*). This manipulation meant that sentences with high-cloze words yielded meanings that were (by definition) consistent with event knowledge, that is, these were stereotypical events. In contrast, sentences inconsistent with real-world experience, (i.e., those with low-cloze words) yielded improbable or unlikely events. As such, conceptual event semantics was an important cue to access and attend to in order to compute meaning coherence.^5^ In contrast, meanings associated with our sentence stimuli with incongruent words consisted of impossible, not improbable events (to paraphrase above example, e.g., *stuffing pillows with #clouds*). As a result, participants did not need to access top-down information regarding stereotypical events in the same way – word by word association and integration was enough to construct a coherent mental representation. As such, the design of our materials did not necessitate a processing strategy associated with accessing global event knowledge – a strategy linked with positive affect (Ashby et al., 1999; Isen and Reeve, 2005; Talarico et al., 2009; Gable and Harmon-Jones, 2010; Selvanayagam et al., 2019). In fact, preliminary evidence suggests that we are on the right track. Given that sentence plausibility is a good measure of conceptual event-based expectations (Matsuki et al., 2011), we note that the off-line plausibility ratings reported above show comparable correlations with negative (*r* = −0.438) and positive (*r* = 0.069) affect. This result supports our preliminary explanation.

## Conclusion

Our work showed a significant N400 effect for sentences such as *The connoisseur tasted the **wine/#roof** on the tour*, which was modulated by dispositional negative affect. This is the first study to show that dispositional affect can modulate N400 magnitude, furthering our understanding of individual differences associated with this component (Tanner et al., 2013; Grey et al., 2017). Furthermore, the findings are consistent with studies in affect and other domains of cognition that suggest a link between global and local perception of stimuli and positive and negative affect. Finally, our work supports views espoused by Münster and Knoeferle (2018), and others that relevant “contextual cues” regarding real-time language processing includes characteristics of the very human being perceiving sentences in context.

## Data Availability Statement

The raw data supporting the conclusions of this article will be made available by the authors, without undue reservation.

## Ethics Statement

The studies involving human participants were reviewed and approved by the Brock University Bioscience Research Ethics Board (BREB) prior to the commencement of the experiment (REB 13-282). The participants provided their written informed consent to participate in this study.

## Author Contributions

VDD conceived and designed the experiment. JS and VDD conducted the experiment, analyzed the data, and wrote the paper. Both the authors contributed to the article and approved the submitted version.

### Conflict of Interest

The authors declare that the research was conducted in the absence of any commercial or financial relationships that could be construed as a potential conflict of interest.

## Footnotes

^1^Note that the ‘#’ symbol (borrowed from semantic theory, cf., Roberts, 1989) indicates that the word does not match its context.

^2^The * symbol is borrowed from syntactic theory, indicating ungrammaticality, as in **He likes Sarah,* where the pronoun *he* lacks an antecedent. We note that using a pronoun or referential noun phrase (e.g., *that wine*) without an explicit antecedent consists of a violation of presupposition and could be thus be equally characterized as a semantic anomaly (as in #). However, for ease of exposition, we use a separate symbol, * for this condition.

^3^20 sentences began with a prepositional phrase (e.g., *After thirty minutes*…). 60 sentences began with a quantifier: (e.g., *Many, Most*). 20 sentences had direct objects as irregular plural nouns, half of which were plural (e.g., *teeth, women*) and half were singular, (e.g., *fireman, mouse*). The remaining 70 sentences were not controlled for any specific sentence construction. These filler items were used in other experiments in our lab.

^4^While the N400 effect was attenuated in the demonstrative condition, as it was still present at electrode CPz, we conducted the correlational analyses here using same ROI. No significant correlations were observed here for either PA, *r*(31) = -0.02, *p* = 0.905, or NA, *r*(31) = -0.01, *p* = 0.967.

^5^Accessibility of this cue was further strengthened since half of all trials contained pseudo-homophones, yielding sentences with no propositional meaning.
